# How far has Africa gone in achieving sustainable development goals? Exploring African dataset

**DOI:** 10.1016/j.dib.2019.104647

**Published:** 2019-10-10

**Authors:** Oluwaseun A. Otekunrin, Siaka Momoh, Idris A. Ayinde, Olutosin A. Otekunrin

**Affiliations:** a Department of Statistics, University of Ibadan, Ibadan, Nigeria; b Department of Agricultural Economics and Farm Management, Federal University of Agriculture, Abeokuta (FUNAAB), Nigeria

**Keywords:** Sustainable development goals index, Extreme poverty, Corruption perceptions index, Stunting

## Abstract

The dataset describes the status of African countries on the attainment of Sustainable Development Goals (SDGs). Datasets on SDG Index (SDGI) scores and other selected variables were compiled from relevant secondary sources. Graphical illustrations were used to describe the type of association existing between the SDGI scores and each of the selected variables to corroborate [1]. Further rigorous statistical analysis can be carried out using these data, in conjunction with other datasets to establish scientifically proven cause and effect relationships among the variables.

**Table:** Specifications Table

Subject	Agricultural Economics
Specific subject area	Food Security, Food Policy
Type of data	Tables, Figures
How data were acquired	Government Implementation Survey
Data format	Raw Filtered Descriptive Analysed
Parameters for data collection	The survey covers the following: (i) National strategies and baseline assessments in the executive (ii) Budgeting practices and procedures in the executive (iii) Stakeholder engagement (iv) Coordinating units in the executive (v) Legislative actions (vi) Main challenges for implementation
Data source location	The datasets explored and analysed are available at: unsdsn.org/resources/publications/2019-africa-sdg-index-and-dashboards-report https://www.transparency.org/files/content/pages/2018_CPI_ExecutiveSummary.pdf https://worldpoverty.io/index.html https://doi.org/10.1016/j.gfs.2019.08.001
Data accessibility	Data is with this article
Related research article	Author's name: Olutosin A. Otekunrin^a^*, Oluwaseun A. Otekunrin^b^, S. Momoh^a^, Idris A. Ayinde^a^ Title: How far has Africa gone in achieving the Zero Hunger Target? Evidence from Nigeria Journal: Global Food Security 22, 1–12 https://doi.org/10.1016/j.gfs.2019.08.001

**Table:** 

**Value of the Data** • These data provide better a clearer picture of why the attainment of SDGs is of great importance on the African continent. • Government, non-governmental organizations, public policy analysts, donor agencies and researchers would benefit from the data. The data provide African countries' progress report on SDGs and other selected indicators that are useful for policy formulation, decision making and advocacy. • Further rigorous statistical analysis can be carried out using these data, in conjunction with other datasets to establish scientifically proven cause and effect relationships among the variables. • These data provide useful cross-country comparisons that can lead to better implementation of the sustainable development goals in Africa.

## 1 Data

Data were compiled from different secondary sources. The 2019 SDGI ranks and scores were extracted from [2], the total population in extreme poverty was gotten from [1,4], the stunting report was obtained from [2] while the Corruption Perceptions Index (CPI) was obtained from [1,3].

Extensive desk research authenticated through consultations with experts and government officials were employed in obtaining the stunting (%) report, SDGI ranks and scores. The SDGI was computed by censoring extreme values from the distribution of each indicator, rescaling the data to enhance comparability across indicators and aggregating the indicators within and across SDGs. The data on total population in extreme poverty were obtained real time using scientifically peer-reviewed and published methodology while the CPI data were obtained from informed views of experts, analysts and businesspeople (in African countries) nationally and internationally.

Table 1 shows the SGDI ranks and scores for each of the 52 countries (Seychelles and Libya are not captured). Tunisia has the highest SDGI score of 66.01 and is ranked 1st in Africa as the highest performer on all SDG goals. South Sudan with the least SDGI score of 29.18 is ranked 52nd in Africa, the worst performer on all SDG goals. Nigeria, South Africa, Egypt, Algeria and Angola are ranked 43rd, 10th, 6th, 3rd and 38th respectively.

**Table 1:** 2019 SDGI ranks and scores.

2019 SDGI Ranks	Country	2019 SDGI Score
1	Tunisia	66.01
2	Mauritius	65.95
3	Algeria	65.55
4	Morocco	64.28
5	Cabo Verde	64
6	Egypt	63.66
7	Sao Tome And Principe	61.78
8	Bostwana	61.44
9	Ghana	61.17
10	South Africa	59.98
11	Gabon	59.06
12	Rwanda	57.9
13	Namibia	57.01
14	Senegal	56.93
15	Kenya	56.53
16	Tanzania	55.94
17	Cote D'ivoire	55.56
18	Uganda	54.88
19	Zimbabwe	54.77
20	Burkina Faso	53.47
21	Ethiopia	53.21
22	Zambia	53.04
23	Togo	52.67
24	Malawi	52.32
25	Eswatini	52.3
26	The Gambia	51.9
27	Mali	51.74
28	Cameroon	51.54
29	Benin	51.48
30	Mozambique	51.4
31	Mauritania	51.25
32	Lesotho	50.84
33	Niger	50.32
34	Burundi	50.25
35	Sierra Leone	49.74
36	Djibouti	49.63
37	Guinea	49.34
38	Angola	49.18
39	Rep. of Congo	48.62
40	Liberia	48.02
41	Comoros	47.5
42	Sudan	47.38
43	Nigeria	47.03
44	Madagascar	45.56
45	Guinea Bissau	45.46
46	Eritrea	43.32
47	Equitorial Guinea	42.06
48	Dem. Rep. of Congo	41.62
49	Somalia	40.12
50	Chad	38.73
51	Central African Rep.	36.7
52	South Sudan	29.18

*Source:* SDG Centre for Africa and Sustainable Development Solutions Network (2019)

**Fig. 1:**
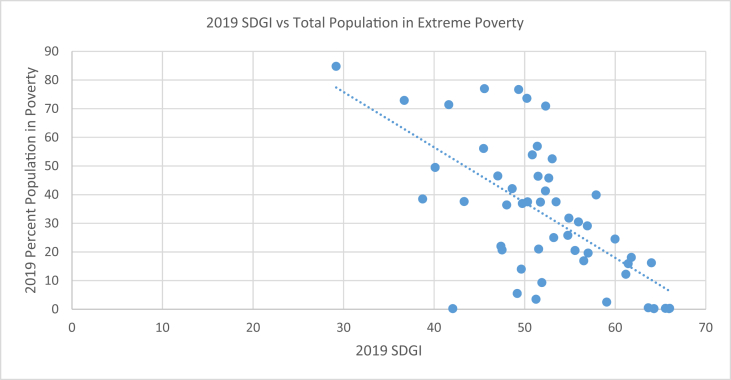
Scatter plot with trend line showing the relationship between 2019 SDGI and total population in extreme poverty (%) of African countries. Source: Authors' graph from compiled data.

**Fig. 2:**
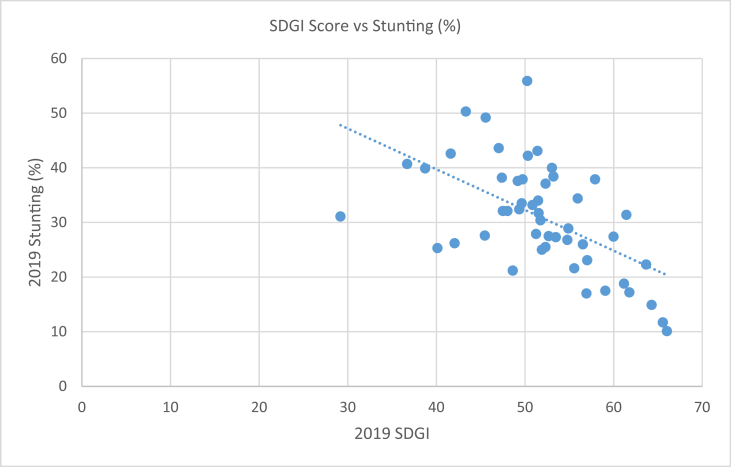
Scatter plot with trend line showing the relationship between 2019 SDGI and stunting (%) of African countries. Source: Authors' graph from compiled data.

**Fig. 3:**
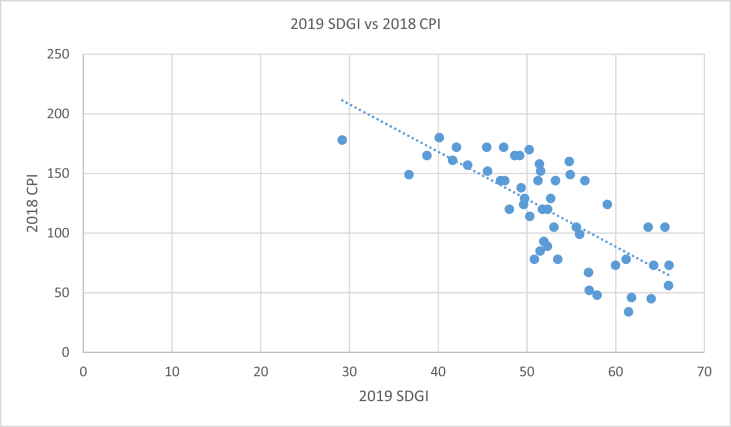
Scatter plot with trend line showing the relationship between 2019 SDGI Score and 2018 CPI Ranks of African countries. Source: Authors' graph from compiled data.

Table 2 compares the SDGI ranks and scores of each country with their respective percentage total population in extreme poverty, percentage population of under 5 stunted children and 2018 CPI Ranks. About 0.3% of the population of Tunisia live in extreme poverty, 10.1% of under 5 children are stunted [5] while the country has a 2018 CPI rank of 73. 84.8% of South Sudan's population live in extreme poverty with 31.1% prevalence of stunting among under 5 children and a 2018 CPI rank of 178. Fig. 1, Fig. 2, Fig. 3 support Table 2 by showing the type of associations existing between the SDGI scores and each of the selected variables.

**Table 2:** 2019 SDGI Ranks and Scores, 2019 percent total population in extreme poverty, 2019 percent population of under 5 stunted children and 2018 CPI Ranks of African Countries.

2019 SDGI Ranks	Country	2019 SDGI Score	Percent Total Population in Extreme Poverty	Stunting (%)	2018CPI Ranks
1	Tunisia	66.01	0.3	10.1	73
2	Mauritius	65.95	0.2	NA	56
3	Algeria	65.55	0.3	11.7	105
4	Morocco	64.28	0.2	14.9	73
5	Cabo Verde	64	16.2	NA	45
6	Egypt	63.66	0.5	22.3	105
7	Sao Tome And Principe	61.78	18.1	17.2	46
8	Bostwana	61.44	15.9	31.4	34
9	Ghana	61.17	12.2	18.8	78
10	South Africa	59.98	24.5	27.4	73
11	Gabon	59.06	2.5	17.5	124
12	Rwanda	57.9	39.9	37.9	48
13	Namibia	57.01	19.6	23.1	52
14	Senegal	56.93	29.1	17	67
15	Kenya	56.53	16.9	26	144
16	Tanzania	55.94	30.5	34.4	99
17	Cote D'ivoire	55.56	20.5	21.6	105
18	Uganda	54.88	31.8	28.9	149
19	Zimbabwe	54.77	25.8	26.8	160
20	Burkina Faso	53.47	37.5	27.3	78
21	Ethiopia	53.21	25	38.4	144
22	Zambia	53.04	52.5	40	105
23	Togo	52.67	45.8	27.5	129
24	Malawi	52.32	70.9	37.1	120
25	Eswatini	52.3	41.3	25.5	89
26	The Gambia	51.9	9.3	25	93
27	Mali	51.74	37.4	30.4	120
28	Cameroon	51.54	21	31.7	152
29	Benin	51.48	46.4	34	85
30	Mozambique	51.4	56.9	43.1	158
31	Mauritania	51.25	3.5	27.9	144
32	Lesotho	50.84	53.9	33.2	78
33	Niger	50.32	37.5	42.2	114
34	Burundi	50.25	73.6	55.9	170
35	Sierra Leone	49.74	36.9	37.9	129
36	Djibouti	49.63	14	33.5	124
37	Guinea	49.34	76.7	32.4	138
38	Angola	49.18	5.5	37.6	165
39	Rep. of Congo	48.62	42.1	21.2	165
40	Liberia	48.02	36.4	32.1	120
41	Comoros	47.5	20.7	32.1	144
42	Sudan	47.38	22	38.2	172
43	Nigeria	47.03	46.5	43.6	144
44	Madagascar	45.56	77	49.2	152
45	Guinea Bissau	45.46	56.1	27.6	172
46	Eritrea	43.32	37.6	50.3	157
47	Equitorial Guinea	42.06	0.2	26.2	172
48	Dem. Rep. of Congo	41.62	71.4	42.6	161
49	Somalia	40.12	49.5	25.3	180
50	Chad	38.73	38.5	39.9	165
51	Central African Rep.	36.7	72.9	40.7	149
52	South Sudan	29.18	84.8	31.1	178

*Source: Authors' compilation from* SDG Centre for Africa and Sustainable Development Solutions Network (2019); Transparency International (2019) and World Poverty Clock (2019)

Note: NA means Not Available.

## 2 Experimental design, materials, and methods

Data on fifty-two (52) African countries were compiled from different secondary sources. The countries were ranked according to their SDGI scores; worst (0) and best (100). Percentage of the total population, in each African country, living in extreme poverty was computed. Prevalence of stunting, in each of these countries, among under 5 year children was measured using the WHO Child Growth Standards. Furthermore, the CPI ranks of these countries were obtained. Graphical illustrations, using Microsoft Excel 2013, were used to describe the type of association existing between the SDGI scores and each of the selected variables.
